# Mortality beyond emergency threshold in a silent crisis– results from a population-based mortality survey in Ouaka prefecture, Central African Republic, 2020

**DOI:** 10.1186/s13031-021-00385-2

**Published:** 2021-06-30

**Authors:** Eve Robinson, Lawrence Lee, Leslie F. Roberts, Aurelie Poelhekke, Xavier Charles, Adelaide Ouabo, Jorieke Vyncke, Cono Ariti, Mariette Claudia Adame Gbanzi, Martial Tanguy Ouakouma, Nell Gray, Maura Daly, Kate White, Sam Templeman, Mia Hejdenberg, Maaike Hersevoort, Sibyl Jade Pena, Anna Kuehne

**Affiliations:** 1 Médecins sans Frontières, Bangui, Central African Republic; 2 Columbia University, New York, USA; 3 Médecins sans Frontières, Amsterdam, The Netherlands; 4 Médecins sans Frontières, London, UK; 5 Centre for Trials research, Cardiff University Medical School, Cardiff, UK; 6 Directorate of Family and Population Health, Ministry of Health and of the Population, Bangui, Central African Republic; 7 Central African Institute for Statistics and Social and Economic Studies, Bangui, Central African Republic; 8 Médecins sans Frontières, Berlin, Germany

**Keywords:** Central African Republic, Mortality survey, Quality of health care, Health services accessibility, Mortality, Maternal mortality, Child mortality, Humanitarian emergency, Violence, Armed conflicts

## Abstract

**Background:**

The Central African Republic (CAR) suffers a protracted conflict and has the second lowest human development index in the world. Available mortality estimates vary and differ in methodology. We undertook a retrospective mortality study in the Ouaka prefecture to obtain reliable mortality data.

**Methods:**

We conducted a population-based two-stage cluster survey from 9 March to 9 April, 2020 in Ouaka prefecture. We aimed to include 64 clusters of 12 households for a required sample size of 3636 persons. We assigned clusters to communes proportional to population size and then used systematic random sampling to identify cluster starting points from a dataset of buildings in each commune. In addition to the mortality survey questions, we included an open question on challenges faced by the household.

**Results:**

We completed 50 clusters with 591 participating households including 4000 household members on the interview day. The median household size was 7 (interquartile range (IQR): 4—9). The median age was 12 (IQR: 5—27). The birth rate was 59.0/1000 population (95% confidence interval (95%-CI): 51.7—67.4).

The crude and under-five mortality rates (CMR & U5MR) were 1.33 (95%-CI: 1.09—1.61) and 1.87 (95%-CI: 1.37–2.54) deaths/10,000 persons/day, respectively. The most common specified causes of death were malaria/fever (16.0%; 95%-CI: 11.0–22.7), violence (13.2%; 95%-CI: 6.3–25.5), diarrhoea/vomiting (10.6%; 95%-CI: 6.2–17.5), and respiratory infections (8.4%; 95%-CI: 4.6–14.8). The maternal mortality ratio (MMR) was 2525/100,000 live births (95%-CI: 825—5794). Challenges reported by households included health problems and access to healthcare, high number of deaths, lack of potable water, insufficient means of subsistence, food insecurity and violence.

**Conclusions:**

The CMR, U5MR and MMR exceed previous estimates, and the CMR exceeds the humanitarian emergency threshold. Violence is a major threat to life, and to physical and mental wellbeing. Other causes of death speak to poor living conditions and poor access to healthcare and preventive measures, corroborated by the challenges reported by households. Many areas of CAR face similar challenges to Ouaka. If these results were generalisable across CAR, the country would suffer one of the highest mortality rates in the world, a reminder that the longstanding “silent crisis” continues.

**Supplementary Information:**

The online version contains supplementary material available at 10.1186/s13031-021-00385-2.

## Background

“*One day, you will come back and you won’t find anyone here because the problems will have killed us all*,” a mother of 11 children told Médecins Sans Frontières (MSF) staff in Ouaka prefecture in Central African Republic (CAR) in August 2018 [1].

CAR has suffered decades of political unrest and conflict, and the people of the Central African Republic are trapped in a cycle of indiscriminate violence [2, 3]. A coup d’état followed by a counterinsurgency in 2013 resulted in more than half a million displaced people and an unknown number of deaths [4]. Episodes of violent attacks subsequently continued across the country [5]. In February 2019, the Khartoum peace deal was signed between the government and 14 armed groups – the sixth peace agreement that has been negotiated since 2013 [6, 7]. However, armed actors continue to commit serious human rights abuses against civilians across the country and more than 70% of the country remains under the control of armed groups [5]. In 2018, CAR was ranked 188^th^ out of 189 countries according to the human development index [8]. It is currently ranked as the 6^th^ most fragile state in the world [9]. CAR has long been referred to as being in a state of “silent crisis”, with many actors pressing that the country has not received the attention or support merited by the difficulties it faces [10, 11]. It is consistently one of the countries that comes furthest from reaching UN funding appeal targets [12].

CAR is one of the lowest ranking countries in the world for a range of health indicators. The average life expectancy of 52.8 years in 2018 is one of the lowest in the world [13]. The median age of the population is estimated at 17.6 years, the second youngest population worldwide [14]. The estimated national maternal mortality ratio (MMR) is the 5^th^ highest in the world at 829 deaths/100,000 live births in 2018 [15]. The estimated birth rate is 35.3 births per 1000 population per year [16], while the infant and neonatal mortality rates are 84 and 41 per 1000 live births respectively, all amongst the highest in the world [17].

The UN estimated a crude mortality rate (CMR) of 12.3/1000/year (equivalent to 0.34/10,000/day) for 2018 [18]. The 2019 and 2018 nationwide SMART surveys estimated higher mortality rates, with a national CMR estimate of 0.84 and 0.73/10,000/day respectively [19, 20]. The same SMART surveys estimated a national under 5 mortality rate (U5MR) of 1.12/10,000/day for 2019 and 0.76 for 2018. A number of regional mortality surveys in 2018 and 2019 estimated varying rates, with some being higher than those estimated by the national SMART surveys and the UN [21–24]. The discrepancy in mortality estimates, and the varying, and sometimes unclear methodologies of these surveys limit their reliability and prevent comparison between areas or over time.

**Fig. 1:**
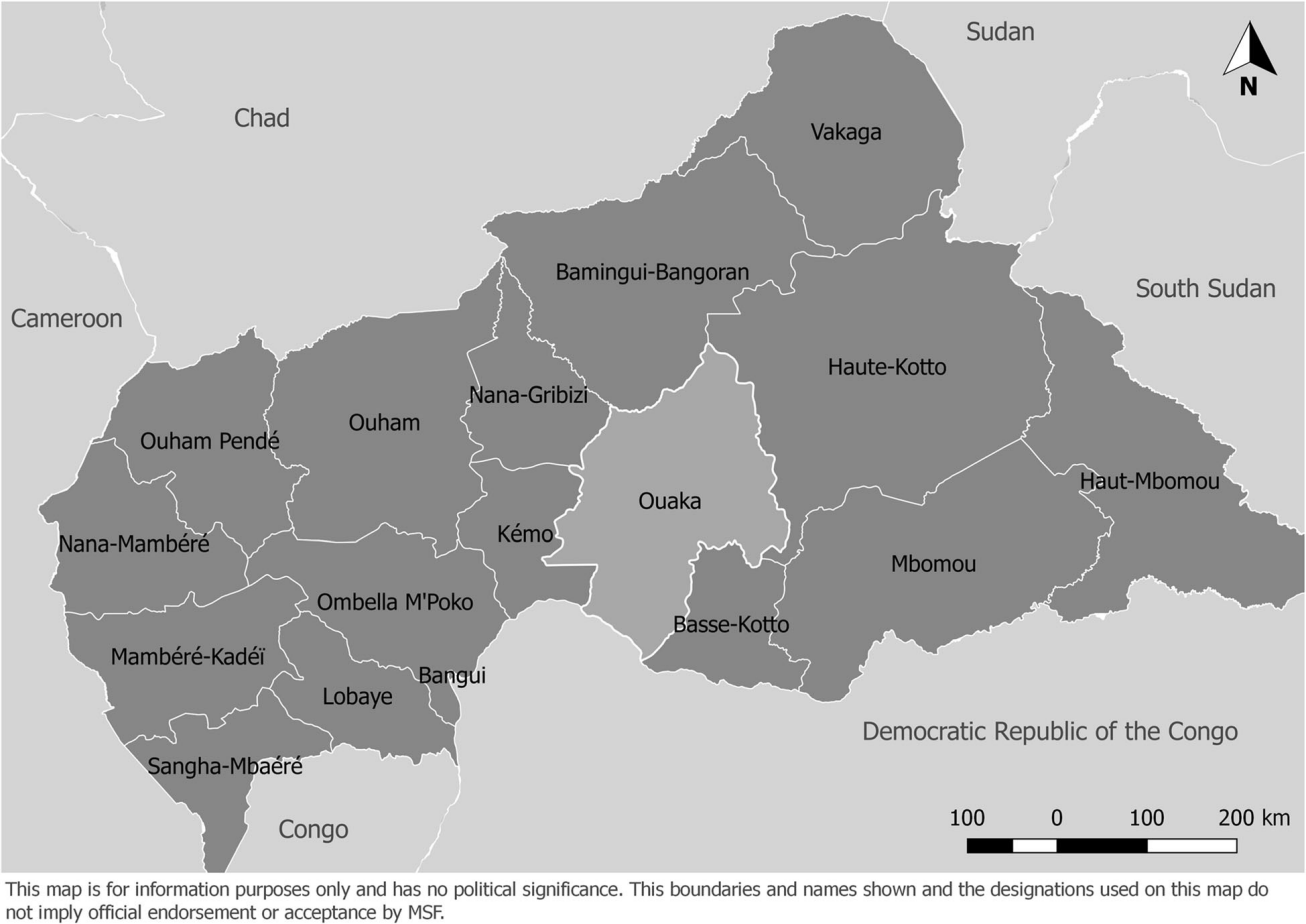
Map of the Central African Republic and its prefectures

Ouaka prefecture (Fig. 1) has an estimated population of 376,821, which represents 7.1% of CAR’s population [25]. The number of internally displaced persons (IDP) in Ouaka decreased from nearly 80,000 in June 2019 to just over 45,000 in April 2020 [26, 27]. Ouaka’s capital, Bambari, has gone through periods of armed conflict since 2014. Prior to a recent upsurge in conflict since mid-December 2020 [28], the last most significant clashes between armed groups took place in January 2019 and resulted in a strong response by the United Nations Multidimensional Integrated Stabilization Mission in the Central African Republic (MINUSCA) [29, 30]. Between then and the recent upsurge in December 2020, Ouaka prefecture had not experienced the same levels of violence as it had before. However, the sustained levels of criminality as well as conflicts between armed actors and attacks on civilians continued, mainly outside the city of Bambari [31].

The healthcare system in Ouaka prefecture includes 32 health posts, 31 health centres, 1 district hospital, 1 secondary hospital, and 1 regional hospital which are fully or partially functional [32]. MSF supports the regional hospital in Bambari, two health centres, one health post, seven points palu (providing care for simple malaria and diarrhoea) and community health promotion activities.

The recent SMART surveys estimated a slighyly lower CMR and U5MR for Ouaka than the national estimate – at 0.77/10,000/day (95% CI 0.47—1.26) and 0.90/10,000/day (0.41—1.96) respectively in 2019 [20], and 0.54/10,000/day (0.32—0.89) and 0.62/10,000/day (0.29—1.34) in 2018 [19].

We conducted a population-based mortality survey in Ouaka prefecture with the objective to estimate the CMR and U5MR, establish causes of death, and estimate the current birth rate and proportion of children < 5 years. Reliable mortality estimates will document the impact of the continued crisis on the health of the community. Knowledge of the most prominent causes of deaths and population structure will allow MSF to plan and prioritise programs accordingly.

## Methods

We conducted a population-based two-stage cluster survey across Ouaka prefecture between 9 March and 9 April, 2020 in cooperation with the Ministry of Health and of the Population and the Central African Institute for Statistics and Economic and Social Studies (*Institut Centrafricaine des Statistique et des Etudes Economique et Sociales* (ICASEES)). The recall period commenced on the 26 May, 2019 (Mother’s Day). We chose this date as it is a well-known celebration in CAR, it fit with our requirement that the recall period cover part of the rainy and the dry seasons, and it was close in time to the end of Ramadan in 2019 (4 June) and to the Catholic feast day of the Ascension (30 May), factors which might further aid recall amongst participants.

Using ENA software for SMART 2011 [33], we estimated a required sample size of 765 households and 3636 persons based on an estimated CMR of 0.72/10,000/day (double the UN estimate available at the time of protocol development [18]), precision of 0.25/10,000/day, design effect (DEFF) of 2, average household size of 5 [21], a non-response rate of 5%, and an average recall period of 265 days. We aimed to sample 64 clusters of 12 households each. The small cluster size was chosen to ensure it was feasible to complete clusters given the expected travel time to some areas, and to minimise time spent stationary in one location due to security risks.

We used two-stage cluster sampling. Cluster sampling is frequently used in settings such as CAR where limited resources, logistical challenges such as poor roads, and security concerns make simple or systematic random sampling methods unfeasible [34–36]. First, we allocated clusters amongst the 16 communes of Ouaka proportional to population size according to population estimates for 2019 from ICASEES [25]. Secondly, we selected buildings as cluster starting points within each commune using systematic random sampling from a dataset of buildings. The sampling frame was created using a dataset of geographical building footprints (based on CAR Ecopia Building Footprint layer,©2019 Digital Globe, Inc) by commune [37, 38]. Buildings in settlements of less than ten buildings were excluded for feasibility reasons. From the cluster starting point, we selected subsequent households in a sequential manner by selecting the next closest building to the right until 12 households were included. We skipped buildings which were not households. In multi-household buildings, we selected one household randomly.

We defined a household as a group who slept under the same roof the previous night, or if a group was spread across several huts, who ate together the previous night. In eligible and consenting households, we included all persons who were members of the household during the recall period.

A priori, we did not exclude any area. We limited the number of clusters that could be replaced to 25%. If the locality of a starting point was deemed inaccessible in advance because of security concerns, we replaced it with another starting point in the same commune as per the stage two sampling previously described. If, on the day of the survey, we could not reach the starting point, or if there was no settlement at the starting point, we replaced the cluster with the next closest village on the return route. If after a second visit a household was still absent, we replaced it. If we did not achieve the target of 12 households after visiting all households in a settlement, we continued in the next closest settlement.

We undertook 4 days of training with the locally recruited survey team on the aim and objectives of the survey, methods, ethics, data protection and smartphone use. During the training, with the assistance of an interpreter, the correct and appropriate phrasing of the survey questions in Sango, the local language, were practiced. We conducted structured interviews (Additional file 1) with the head of household or a designate. The head of the household (or designate) could be any adult member of the household who could provide information on events in the household over the recall period. Households self-identified the head of the household. We started the survey with an open question about challenges the household faces (“What difficulties does your household and community face on a daily basis?”) both to build rapport and to document general difficulties in the community. We noted the responses or a summary on paper. Then, using KoBoToolbox [39] on smartphones, we asked a series of questions on household composition during the recall period. For all identified members of the household during the recall period we asked demographic information, and noted arrivals or departures. For women aged 10–49 years we also asked about pregnancy during the recall period, and the outcome of the pregnancy. For deaths, the reported cause and place of death, and health seeking behaviour in the 2 weeks prior to the death was recorded. For the cause of death, the household was asked an open question and allowed to respond freely. If the response corresponded with one of the pre-defined categories listed in the questionnaire, (see Additional file 1) we marked this. If not, we noted as free text the reported cause of death or any additional information provided. The epidemiologist (ER who is a medical practitioner) reviewed these responses, and if sufficient information was available, categorised the cause of death.

The recall period ran from 26 May, 2019 to the interview date. For members who left/died or arrived/were born during the recall period, their person-time contribution was adjusted for the exact date of the event if known. Otherwise, the mid-month was used.

Using Poisson regression, we calculated the CMR and U5MR as deaths/10,000 population/day, the MMR as maternal deaths/100,000 live-births, and the neonatal mortality rate as deaths in the first 28 days of life/1000 live-births. We categorised the outcome of pregnancy as live-born, early pregnancy loss (< 3 months gestation or before a woman was visibly pregnant), late pregnancy loss (> 3 months or after a woman was visibly pregnant, including stillbirths). While we did not specifically ask if a pregnancy loss was a spontaneous or induced abortion, if the household mentioned it was an induced abortion this was noted.

We present descriptive analyses as proportions with 95% CIs for categorical variables and means and standard deviations (SD) or medians including interquartile ranges (IQR) for continuous variables. Where appropriate, we measured differences in proportions using Pearson χ2 test and present a *p*-value (p).

All analyses were conducted accounting for the survey sample weights and the effect of clustering induced by the two-stage sampling method. We undertook quantitative data analysis using Stata version 15.1 [40].

We digitalised the qualitative data collected in response to the introductory questions on difficulties faced. We coded it using a content analysis approach in order to identify themes and patterns from the data [41, 42]. We describe the identified themes and include a selection of illustrative quotations. Of note, during the first 2 days of interviews the responses of each individual household were not documented.

Of note, we planned to conduct six other surveys using the same methodology across CAR – four studies in the other prefectures where MSF is present (Ouham, Mambere-Kadei, Mbomou, and Haute Kotto); one in the capital region of Bangui; and one covering all other prefectures. We planned to merge and weight the results of each study to provide country-wide estimates. The studies were due to take place in a staggered fashion in the first half of 2020. Ouaka was the first prefecture to start and was almost complete when the first case of COVID-19/ Sars-CoV-2 was detected in CAR. Unfortunately, the other surveys, which had not yet started, could not proceed for a number of reasons including travel restrictions to and within CAR, redirection of resources towards the COVID-19 response, and uncertainty regarding the impact of COVID-19 on the security context in CAR.

## Results

**Fig. 2:**
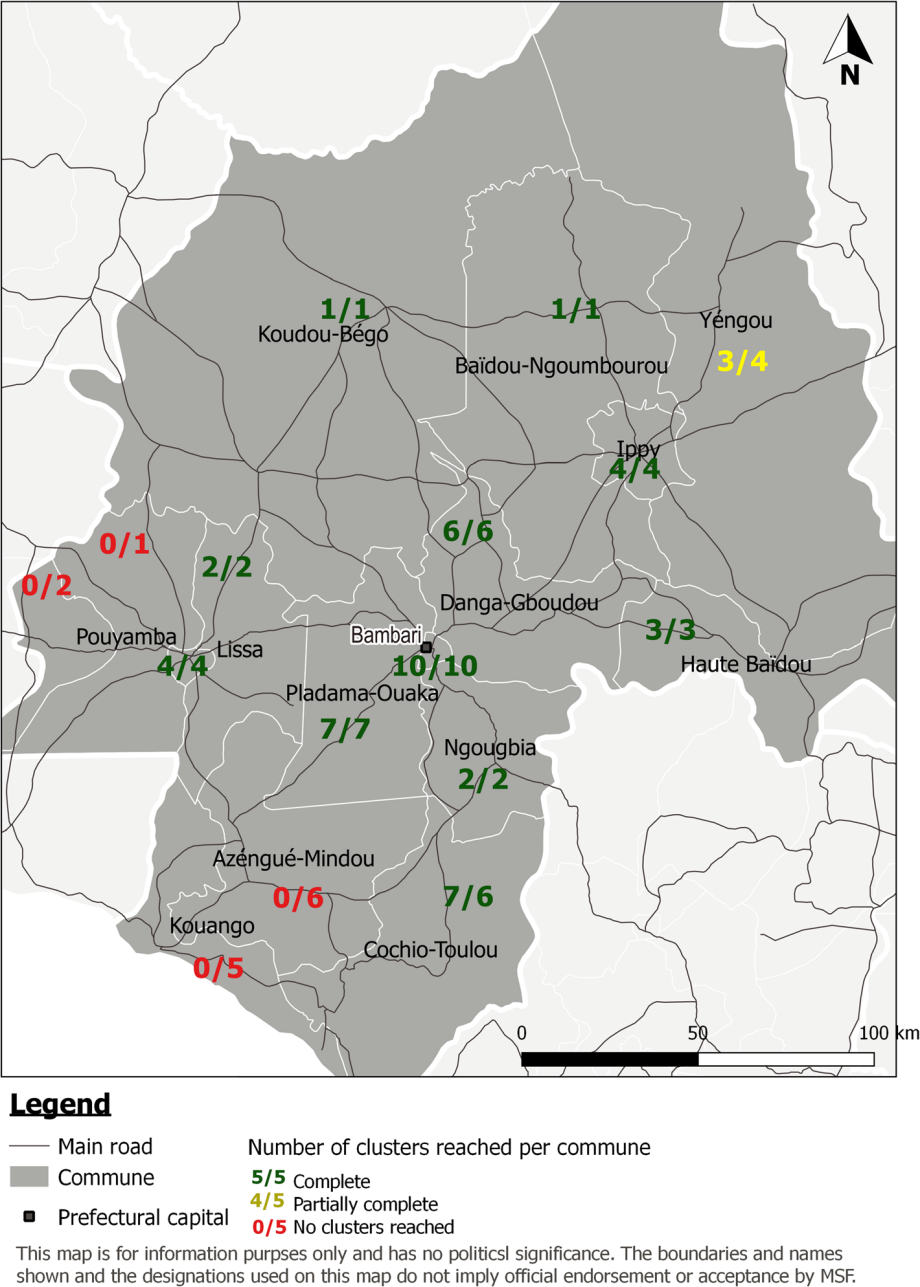
Number of completed clusters for each commune of the prefecture of Ouaka, Retrospective mortality study, Ouaka, CAR, 2020

We completed 50 clusters across 12 communes (Fig. 2). Of the initial 64 clusters sampled, we discarded 21 due to insecurity and/or inaccessibility. A replacement cluster was accessible for seven of the discarded clusters. We did not complete any points in 4 of the 16 communes in the prefecture of Ouaka: Azengue-Mindou, Kouango, Pouyamba and Kobadja, due to insecurity in the commune or along the access route.

For 3 cluster starting points, no inhabited building could be found upon arrival at the point. We replaced these with the next nearest village passed.

We visited 693 households of which 93 were absent (13.4%) and 9 refused to participate (1.3%); of 600 households present, 591 (98.5%) participated. In the included households, there were 4272 household members during the recall period and 4000 household members on the interview day. There were 198 household members born and 14 who joined the household, while 160 died and 112 left the household during the recall period (Table 1). The median household size was 7 persons (IQR 4–9; mean 7.2 with 95% CI: 6.6–7.7).

**Table 1:** Arrivals and departures of included household members during the recall period, Retrospective mortality survey, Ouaka, CAR, 2020

Status during recall period	N	
Present at start		4060
Arrivals		212
Births	198	
Other arrival	14	
Departures		272
Deaths	160	
Other departure	112	
Present at end (during the interview)		4000

### Study population

**Fig. 3:**
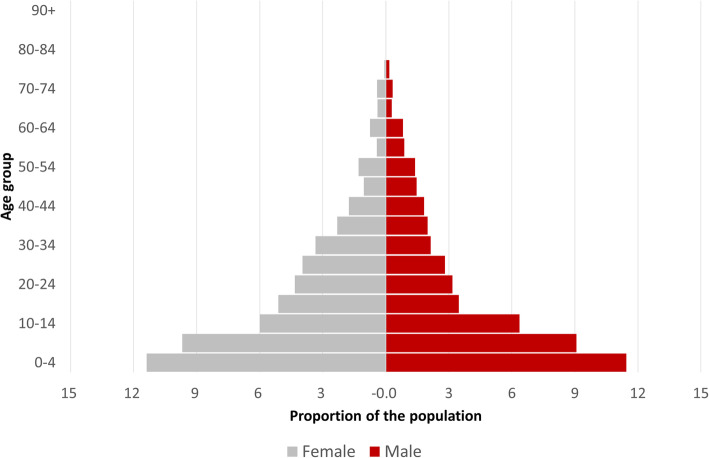
Age distribution of population by five-year age groups and by gender; Retrospective mortality survey, Ouaka, CAR, 2020

The median age of the population was 12 years (IQR 5—27); 14 years (IQR 5—27) for females and 12 (IQR 5—28) years for males. The mean age was 18.1 years (95% CI: 17.6—18.7); same for males and females (18.1 years; 95% CI: 17.4—18.9 for both). Females contributed to 52.4% (2223/4272; 95% CI: 50.8—54.0%) of the population. Children under-five made up 22.8% (969/4270; 95% CI: 21.4—24.3%) of the population (Fig. 3 and Table 2). Persons aged 60 years or over made up 3.3% (144/4270; 95% CI: 2.7—4.0%) of the population.

**Table 2:** Child and adult populations, by gender (number and weighted proportions; *n* = 4272); Retrospective mortality survey, Ouaka, CAR, 2020

Age	All participants		Females		Males		Female to male ratio
	n	%	N	%	n	%	
0—4 years	969	22.8	477	11.4	492	11.5	1.0
*< 12 months*	*215*	*5.1*	*105*	*2.5*	*110*	*2.6*	
*0—2 months*	*61*	*1.4*	*31*	*0.7*	*30*	*0.7*	
*3—5 months*	*62*	*1.5*	*35*	*0.8*	*27*	*0.7*	
*6—8 months*	*57*	*1.3*	*23*	*0.6*	*34*	*0.8*	
*9—11 months*	*35*	*0.9*	*16*	*0.4*	*19*	*0.5*	
*12—23 months*	*147*	*3.4*	*63*	*1.5*	*84*	*1.9*	
*24—35 months*	*201*	*4.8*	*104*	*2.5*	*97*	*2.3*	
*36—47 months*	*211*	*5.0*	*100*	*2.4*	*111*	*2.6*	
*47—59 months*	*195*	*4.6*	*105*	*2.6*	*90*	*2.1*	
5—9 years	803	18.8	415	9.7	388	9.1	1.1
10—14 years	527	12.4	255	6.0	272	6.4	0.9
14—19 years	371	8.6	217	5.1	154	3.5	1.5
20—24 years	324	7.5	183	4.3	141	3.2	1.4
25—29 years	288	6.8	166	4.0	122	2.8	1.4
30—34 years	232	5.5	140	3.4	92	2.1	1.6
35—39 years	186	4.4	100	2.3	86	2.0	1.2
40—44 years	149	3.6	72	1.8	77	1.8	1.0
45—49 years	108	2.5	46	1.1	62	1.5	0.7
50—54 years	112	2.7	54	1.3	58	1.4	0.9
55—59 years	57	1.3	20	0.4	37	0.9	0.5
60—64 years	66	1.6	33	0.8	33	0.8	0.9
65—69 years	29	0.7	17	0.4	12	0.3	1.4
70—74 years	34	0.7	20	0.4	14	0.3	1.3
75—89 years	11	0.3	4	0.1	7	0.2	0.4
80+ years	4	0.0	3	0.0	1	0.0	2.3
Unknown	2		1		1		
Total	4272		2223	52.4	2049	47.6	1.1

### Pregnancy and birth

Amongst the total population present on the day of the interview, 3.0% (120/4000; 95% CI: 2.5—3.5) were currently pregnant. The proportion of females aged 10 to 49 years who were pregnant during the recall period was 29.1% (346/1179; 95% CI: 26.4—31.9%). The median age of females who were pregnant at any stage during the recall was 23 years (IQR 19—29 years). Four females who were pregnant were under the age of 15, with the youngest being 12 years of age.

Of the 220 reported pregnancies which ended during the recall period, 87.1% (194/220; 95% CI: 80.2—91.9%) resulted in a live-birth (199 neonates due to five sets of twins); 5.8% (13/220; 95% CI: 3.1—10.7%) ended in early pregnancy losses and 5.3% (10/220; 95% CI: 2.7—10.1%) in late pregnancy losses and an additional four cases (1.8%, 4/220; 95% CI: 0.4—7.8%) were reported as induced abortions. We estimated the crude birth rate at 59.0 births per 1000 population per year (95% CI: 51.7—67.4).

### Mortality and causes of death

A total of 160 deaths were reported during the recall period. Of these, 29.4% (47/160; 95% CI: 22.4—37.4%) were in children under-five, and 59.2% (96/160; 95% CI: 49.3—68.3%) were in males.

The CMR was 1.33 deaths/10,000 persons/day (95% CI: 1.09—1.61), equivalent to 48.4 deaths/1000/year. The U5MR rate was 1.87 deaths/10,000 persons/day (95% CI: 1.37—2.54), and the neonatal mortality rate was 32.0/ 1000 births (95% CI: 14.6—70.1).

There was not a statistically significant difference in the CMR over different seasons of the recall period. From 26 May, 2019 (start of recall period) to 31 August, 2019, the CMR was 1.16 (95% CI: 0.84—1.61); from 1 September, 2019 to 31 December, 2019, the CMR was 1.11 (95% CI: 0.84—1.48); and from 1 January, 2020 to the end of the survey, the CMR was 1.46 (95% CI: 1.1—1.91).

Overall, the most frequently specified causes of death reported were fever/malaria (26/160; 16.0%; 95% CI: 11.0—22.7%), violence (22/160; 13.2%; 95% CI: 6.3—25.5%), and diarrhoea/vomiting (16/160; 10.6%; 95% CI: 6.2—17.5%) (Table 3). There was no evident seasonality of reported causes of death (data not shown).

**Table 3:** Causes of death by age group (number and weighted proportions), Retrospective mortality survey, Ouaka, CAR, 2020

Cause of death	Total		< 5 years		≥5 years	
	n	% (95% CI)	n	% (95% CI)	n	% (95% CI)
Malaria/fever	26	16.0 (11.0—22.7)	15	30.5 (17.8—47.1)	11	9.9 (5.9—16.2)
Violence	22	13.2 (6.3—25.5)	2	4.8 (0.6—28.8)	20	16.7 (7.7—32.5)
Diarrhoea/vomiting	16	10.6 (6.2—17.5)	11	24.0 (11.9—42.7)	5	5.0 (1.9—12.2)
Respiratory infection	13	8.4 (4.6—14.8)	3	6.8 (2.1—20.1)	10	9.1 (4.6—17.1)
Neonatal	6	3.5 (1.6—7.5)	6	11.9 (5.3—24.7)		
Maternal	5	3.1 (1.4—6.8)			5	4.4 (2.0—9.4)
Measles^a^	4	2.6 (1.0—6.9)	3	6.6 (2.1—19.0)	1	0.9 (0.1—6.7)
Trauma/accident	3	2.3 (0.7—7.3)	1	2.3 (0.3—16.2)	2	2.3 (0.5—9.3)
Malnutrition	2	1.3 (0.3—4.9)	1	2.4 (0.3—16.5)	1	0.9 (0.1—6.0)
Other – specified^b^	15	9.3 (5.4—15.3)	0		15	13.1 (7.7—21.3)
Unknown	48	29.9 (21.7—39.6)	5	10.6 (4.2—24.3)	43	37.9 (26.6—50.6)
Total	160		47		113	

^a^One additional death in a child < 5 years was reported as having measles and malaria. The cause of death has been classified as malaria/fever for the analysis

^b^Includes: hernia (*n* = 4), 2 of which died post a surgical intervention; typhoid (*n* = 4); diabetes (*n* = 2); asthma (*n* = 1); snake-bite (*n* = 1); mumps (*n* = 1); meningitis (*n* = 1); and an abscess (*n* = 1))

Violence was the most common specified cause of death among participants ≥5 years, accounting for 16.7% (20/113; 95% CI: 7.7—32.5) of deaths. Males accounted for 20 of the 22 violent deaths. Violent deaths were reported by 16 households across 11 clusters and 7 communes: Bambari (*n=*10), Yengou (*n=*4), Cochio-Toulou (*n=*3), Danga-Gboudou (*n=*2), Lissa (*n=*1), Grimari (*n=*1), and Baidou- Ngouboudou (*n=*1). We did not ask specific questions relating to violent deaths, but when additional information was provided, the perpetrators were reported as armed groups (*n* = 13), bandits (*n* = 3), and a spouse (*n* = 1). The type of injuries inflicted were reported as gunshots (*n* = 5; specifically reported to have been caught in cross-fire in 4 cases), slashing of throat (*n* = 3), stabbing (*n* = 1), gunshot and slashing of throat (*n* = 1), and being pulled along the roadside by a vehicle (*n* = 1). For 11 cases it was reported that the victim was attacked or killed, but the exact method was not reported. The violent deaths of two siblings occurred together and were related to a conflict in a neighbouring prefecture. The violent deaths of 4 members of the same household occurred together when they are reported to have been attacked by bandits as they travelled as part of the transhumance (the seasonal movement of pastoralists with their livestock). Otherwise, violent deaths were not clustered in time or related to known armed offensives.

For 48 deaths a specific cause was not identified. For 44 of these, the household could describe symptoms or the circumstances which preceded the death. However, these could not be reliably attributed to a specific cause (e.g. ‘vomiting blood with cough’; ‘swelling of legs, diarrhoea, weight-loss for several months’) or the reported cause would not itself explain the death (e.g. sorcery). (See Additional file 2).

Five maternal deaths were identified – one following an early pregnancy loss and was preceded by abdominal pain and fever; one occurred intrapartum in hospital; one occurred postpartum following a procedure to clear the uterus; and two followed an abortion induced by taking medicines. Amongst women of reproductive age, 13.9% (5/34; 95% CI: 6.8—26.2) of deaths were maternal. The MMR was 2525/100,000 live-births (95% CI: 825—5794).

### Healthcare seeking behaviour

Amongst those who died, 62.0% (99/160; 95% CI: 53.2—70.0%) reportedly sought care in a healthcare facility in the 2 weeks preceding death (Table 4). There was not a statistically significant difference in the proportion seeking care in a healthcare facility between males and females (61.9% vs 62.1%; *p* = 1.0) or between those < 5 years and ≥ 5 years (66.9% vs 60.0%; *p* = 0.4).

**Table 4:** Reported health seeking behaviour amongst those that died (*N* = 160), Retrospective mortality survey, Ouaka, CAR, 2020

	Total		< 5		≥5	
	n^a^	% (95% CI)	n^a^	% (95% CI)	n^a^	% (95% CI)
Healthcare facility	99	62.0 (53.2—70.0)	32	66.9 (51.0—70.7)	67	60.0 (50.0—69.1)
*Community health Facility*	*41*	*27.1 (18.1—38.4))*	*19*	*42.5 (26.6—60.1)*	*22*	*20.7 (21.2—32.9))*
*Health centre*	*13*	*7.1 (3.3—14.5)*	*4*	*8.1 (2.3—25.3)*	*9*	*6.6 (3.2—13.2)*
*Health post*	*28*	*20.0 (12.4—30.9)*	*15*	*34.3 (21.2—50.3)*	*13*	*14.1 (7.1—25.9))*
*Hospital*	*59*	*35.3 (26.4—45.3)*	*13*	*24.5 (13.5—40.3))*	*46*	*39.8 (29.9—50.5)*
Self-medication	9	5.3 (2.9—9.7)	2	4.3 (1.1—15.2)	7	5.7 (2.8—11.5)
Traditional practitioner	7	4.7 (2.2—9.9)	1	3.0 (0.4—19.9)	6	5.4 (2.3—12.5)
No care sought	46	28.7 (20.3—38.8)	12	25.6 (15.0—40.5)	34	29.9 (20.1—42.0)
Total	160		47		113	

^a^more than one option could be noted

All cases of maternal deaths (*n* = 5) and malnutrition (*n* = 2) sought healthcare in the 2 weeks preceeding their deaths and more than two-thirds of cases who died of measles, malaria, respiratory infections and diarrhoea did so.

In all cases where healthcare was not reported to have been sought in a healthcare facility prior to the death (61/160; 38.0%; 95% CI: 30.0—46.8), the most common reasons for not seeking healthcare were immediate death or very short illness (*n* = 30), followed by a lack of money for consultations (*n* = 15), self-medication (*n* = 8), visiting a traditional healer (*n* = 7), security concerns (*n* = 3), and distance to a healthcare facility (*n* = 3).

### Challenges reported by the households

Responses to the open question on challenges the household faced in the past year were documented for 520 of 591 participating households.

The most common theme was related to health and healthcare (378 households). The households described a high incidence of specific illnesses, in particular the burden of gastrointestinal illnesses: “*Diarrhoea, vomiting is widespread in this household”* [household 172, cluster 15, Danga-Gboudou commune]; malaria: “*The children have malaria constantly*” [household 231, cluster 21, Pladama-Ouaka commune]; and measles: “*We suffer a lot from measles*” [household 575, cluster 71, Ippy commune].

Difficulties in accessing healthcare due to either absence of healthcare facilities or distance were frequently cited (64 households): “*…it is necessary to go to Bambari for treatment*” [household 168, cluster 14, Pladama-Ouaka commune]; as were financial barriers to accessing care or medication (34 households): *“We have a health centre but that requires money, we don’t have money”* [household 244, cluster 22, Pladama-Ouaka commune]. Lack of medicines and lack of (trained) staff in the healthcare facilities were described often (23 households): “*We have a health centre which doesn’t have enough medicines”* [household 251, cluster 22, Pladama-Ouaka commune]*; “The centre does not have all the means to save lives here*” [household 553, cluster 70, Cochio Toulou commune].

Challenges related to reproductive health were mentioned by 14 households, including spontaneous and induced abortion, stillbirths, infertility, illness during pregnancy and access to maternal health care: “*The women have serious problems while they are pregnant*” [household 514, cluster 57, Cochio Toulou commune]; “*Care of pregnant women is not available at the centre which occasionally leads to the deaths of some pregnant women on the site”* [household 282, cluster 25, Pladama-Ouaka commune]*.*

Challenge related to deaths and bereavement were not uncommon (67 households). Several households spoke generally about a high number of deaths in the family or the village: “*Too many deaths in our household given this crisis*” [household 108, cluster 9, Bambari commune]; while others spoke of the deaths family members, including outside the recall period. Households spoke of difficulties in looking after children after the death of a parent: “*Lots of grandchildren to look after and I don’t have the means to look after them properly. Their mother and father died 2 or 3 years ago*” [household 55, cluster 5, Bambari commune]; “*My brother died and his children are my responsibility*” [household 356, cluster 36, Ippy commune]. Several households were headed by widows, and other households spoke of the number of widows in the community: “*After the crisis there are a lot of widows*” [household 131, cluster 11, Danga-Gboudou commune].

Problems related to insecurity or conflict were common (130 households), mainly related to armed groups. Across nearly all interviews there were references to the conflict, referred to as “*the events*”, “*the crisis*”, and “*the conflict*”. Many households spoke of deaths related to conflict including deaths outside of the recall period: “*I lost my wife and my 3 month old child due to crossfire*” [household 326, cluster 30, Ippy commune]. Households spoke of stress caused by conflict related deaths: “*Too many cases of death in our household given this crisis; too much stress given these cases of death”* [household 108, cluster 9, Bambari commune]. Households described the general menace to the civil population caused by the presence of armed groups and displacement due to security: “*We have to move every year*” [household 426, cluster 44, Lissa commune]. They also recounted how security issues limit their movements and the consequences of this: “*We can’t go to the fields*” [household 187, cluster 16, Danga-Gboudou commune]; “*Roadblocks for the sick to go to the health centre which caused the death of my wife and children*” [household 201, cluster 17, Haute Baidou commune]. Some households reported taxes or fines imposed by armed groups on households and villages. Several households recounted specific incidents perpetrated on a household member by armed groups: “*She sells alcoholic drinks but when the armed groups arrive, they take it and don’t pay*” [household 163, cluster 14, Danga-Gboudou commune]. Intra-familial conflict and missing household members related to the conflict were also mentioned. Problems related to theft and lootings, in particular armed thefts, were also mentioned. Several households reported having been plundered, and their houses being burnt down, in the past year: “*They burnt my house, I lost everything*” [household 579, cluster 72, Yengou commune].

Themes relating to poor living conditions were also common. A lack or absence of clean drinking water was frequently mentioned (198 households): “*To access water we must go 3km into the bush*” [household 541, cluster 66, Pladama-Ouaka commune]. Households spoke about the dirty water and linked the lack of clean water to the gastrointestinal illnesses they suffer: “*The lack of potable water means we drink backwater which results in stomach aches, intestinal worms*” [household 182, cluster 16, Danga-Gboudou commune]. Problems related to food insecurity (139 households): “*We live on only wild yams*” [household 131, cluster 11, Danga-Gboudou commune]; financial or other difficulties in means of subsistence (151 households): “*we have money difficulties*” [household 322, cluster 29, Ippy comune]; lack of non-food items including bednets (49 households): “*following the events we were looted”* [household 578, cluster 72, Yengou commune]; and lack of shelter were other common themes: (20 households): “*we don’t have a tarpaulin for our roof*” [household 455, cluster 52, Koudou-Bego commune]*.*

## Discussion

Our survey showed a very young population with a high birth rate that is experiencing a high number of deaths affecting all members of society: adults, children and mothers. The median age was substantially younger than previous estimates at 12 years, while the mortality rates far exceeded previous estimates and the CMR crosses the threshold for an acute humanitarian emergency of 1 death/10,000 persons/day [18–20, 34, 43, 44].

Our estimate of the CMR in Ouaka for the period between May 2019 and April 2020 of 1.33 deaths/10,000 persons/day (95% CI: 1.09—1.61) is higher than other recent estimates for Ouaka, and approximately 4-times the UN national estimate of 0.34 deaths/10,000/day [18–20]. A possible reason could be that previous surveys included additional items such as malnutrition assessments and there is some evidence that mortality-only surveys, such as this one, might improve mortality estimates [45, 46]. In addition to the high CMR, deaths and their psychological and social impact was a strong theme emerging from the responses to the open question on challenges experienced by the household. Many households spoke of deaths which fell outside of the recall period, or the difficulties faced by being a widow or caring for orphans, suggesting a persistently high mortality rate.

We found violence to be the most common specified cause of deaths in those ≥5 years of age, responsible for 16.7% (20/113; 95% CI: 7.7—32.5) of deaths. An MSF survey in the neighbouring area of Alindao (Basse-Kotto prefecture) in 2019, found a similarly high proportion (15%) of violence-related deaths, albeit after an armed offensive in the area [21]. The large proportion of violence related deaths is particularly notable as there was not widespread or prolonged conflict across Ouaka during the recall period. Violence related deaths were reported in 7 out of 12 communes reachable for the survey, including Bambari town, an apparent “weapon free” zone with a strong presence of peace-keeping forces [47]. In addition, some areas, were not reached by the study due to localised security concerns. Therefore, violence related deaths may in fact be underestimated. It is also notable that most of the violence related deaths appeared to be single acts of violence rather than part of a particular offensive of armed groups. Correspondingly, in response to the open question, households across the prefecture spoke of the menace posed by armed groups, and how conflict, or the fear of violent attacks impacts on their daily lives. Incidents of non-fatal attacks were also reported.

The U5MR (1.87 deaths/10,000 persons/day (95% CI: 1.37—2.54)) rests just below the emergency threshold of 2 deaths/10,000 persons/day [34, 43, 44]. The upper confidence limit extends beyond the threshold, suggesting the U5MR may meet the emergency threshold. The U5MR is twice as high as that previously reported for Ouaka [19, 20]. Underreporting of child deaths, particularly neonatal deaths, during household surveys has been reported previously [45, 46, 48]. We started with an open question to build rapport with households, potentially making it easier for the household to discuss distressing or possibly stigmatising events such as the death of a child. In addition, our surveyors received specific training on the usual course of pregnancy and birth as well as asking sensitive questions about the outcome of birth. These features may have enabled us to capture more neonatal deaths than the previous SMART surveys and partially explain the difference in estimates. Another explanation may be because the SMART surveys in both years were conducted between September and October/December with a 90-day recall period and therefore may not have included the entire peak malaria season [19, 20].

Of deaths in children < 5 years, 64% were caused by three common and treatable illnesses: malaria, diarrhoea and respiratory infections. In addition, and unsurprisingly given a known measles outbreak in Ouaka during the recall period, measles was the 4^th^ most common cause of death for children < 5. In response to the open question, households also spoke about a high incidence of these illnesses amongst children. The households additionally spoke of living conditions which would contribute to these illnesses such as lack of access to potable water and bednets Many households also spoke of barriers to accessing care including the absence of a healthcare facilities, distance, and cost.

CAR is estimated to have the 5^th^ highest MMR in the world at 829 deaths/1000,000 live-births [15]. Our MMR estimate for Ouaka is considerably higher at 2525/100,000 live-births. While the lower confidence limit (825 deaths/100,000 live-birth) of our estimate is similar to the UN estimate, the upper confidence limit extends to 5794 deaths/100,000 live-birth, suggesting the true MMR in Ouaka could be up to seven times higher than the UN estimate for CAR [15]. Our MMR estimate indicates that Ouaka might be among the deadliest places for pregnant women globally. The estimated MMR needs to be interpreted with caution as the sample size was not powered to provide a precise estimate. However, we also found a high neonatal death rate and a high birth rate, both linked to maternal health and mortality. Poor health during pregnancy and difficulty in accessing care while pregnant was also reported by a several households in response to the opening question. Notably, two of the five maternal deaths were reported to have occurred following an induced abortion. Complications from unsafe abortions are responsible for 18% of maternal mortality in Africa [49]. While we did not delineate between safe and unsafe abortions, given the restrictive legal framework in CAR we can assume that the availability safe abortion care in this region is limited. Our findings indicate the need for improved access to sexual and reproductive health services, including safe abortion and post abortion care.

The mortality estimates of this study are considerably higher than recent UN estimates for CAR which raises questions about the reliability of the UN health statistics, and their underlying, sometimes outdated, data sources. Various UN mortality estimates for CAR cite sources for their modelled estimates including the 2003 census, the 2010 MICS and the 1995 DHS [15, 50–52] i.e. the latest source being more than a decade old and pre-dating the start of the conflict in 2013. The recent 2019 MICS estimated that 99 of 1000 children born die by their 5^th^ birthday [53], which is in fact lower than the UN estimate for 2019 of 110. However, both estimates approximate to only a fraction of U5MR reported by this and other recent studies [19–21, 54]. Surveys with many components such as MICS, and surveys with long recalls can grossly underestimate mortality, especially in this region [45, 46, 55]. Estimates of mortality which are modelled from national data which is outdated, of low-quality, or not representative (due to the exclusion of inaccessible areas) may not reflect the true situation, particularly in unstable settings such as CAR, and therefore should be interpreted with caution. Well designed and implemented population-based mortality surveys can provide more reliable data in such settings [56].

Regarding the population structure, we found a younger median age and higher proportion of children < 5 years than estimated by the UN [14]. Two recent population-based surveys in other prefectures estimated a similarly high proportion of children < 5 years: 21.1% in in Alindao (Basse-Kotto) in 2019 [21], and 23.2% in Ouham Pendé in 2018 [57]. The 2019 MICS survey, estimated 20.2% of the population was < 5 years old [53]. Similarly, the birth rate we estimated is higher than the UN estimate [16]. There are no reliable civil registration data available to monitor birth or death rates in CAR. A population-based surveillance system implemented by MSF in Lobaye prefecture found a similar birth rate of 61.8/1000 person-years (95% CI: 54.7—6 9.9) in 2010 [58]. This potential underestimation of the birth rate may indicate an underestimation of population growth, which in turn may underestimate child and maternal care needs, vaccination coverage denominators and other crucial indicators for population health.

The reasons why many who sought healthcare still died was not explored. While a desirability bias may have caused over-reporting of healthcare seeking, other possibilities include: delays in seeking care; barriers to adhering to treatment; or because the care available was inadequate or of poor quality. Notably, very few households mentioned seeking care at a traditional practitioner and many reported seeking care at a health facility, which might also indicate some desirability bias. In response to the open question, some households questioned the quality of care available, speaking of a lack of medicines, healthcare workers or other means in healthcare facilities. It is increasingly recognised that increasing access to care and utilisation of services is not sufficient to improve child mortality if the care available is of poor quality [59].

The responses to the open question provided an insight into the challenges faced by the community and their impact on health. While health problems and access to healthcare was the predominant theme to emerge, other challenges were evident. Many of these have a clear direct impact on health such as food insecurity and access to potable water, as manifested by the leading contribution of diarrhoeal disease to death in children While the high proportion of deaths related to violence detected in the mortality questionnaire does demonstrate the direct impact of conflict on health, it misses the long lasting psychological and social impacts which are evident from the responses to the open question. Other challenges reported, such as lack of shelter, non-food items and means of subsistence, are major contributors to the wider determinants of health. It is a reminder that improving health requires actions far wider than the provision of healthcare.

In response to these results, the MSF project in Ouaka will continue to focus on child health programmes, in particular the prevention and management of the main preventable and easily treatable illnesses. One method of doing this is the expansion of the coverage of integrated community case management (iCCM) to improve basic but quality healthcare for malaria, diarrhoea and respiratory infections in hard-to-reach areas. Furthermore, MSF will increase easily accessible basic pregnancy care and explore the magnitude and causes of maternal deaths further.

In addition to supporting operational decisions, this study aimed to document the impact of the continued crisis on the health of the community and to advocate for more comprehensive support for the country. Decades of political instability, limited development of structures, and cyclical conflict problems are inextricably and symbiotically linked, ultimately resulting in highly vulnerable communities [60]. Despite the hope arising from the 2019 peace agreement, the security and humanitarian situation across CAR had deteriorated in 2020 [61, 62]. A recent upsurge in violence across CAR has led to an even further deterioration, with over 276,000 people displaced since mid-December 2020, widespread disruption of supply chains into and around CAR, and violations of basic international humanitarian law principles, including an armed offensives in Ouaka which damaged an MSF supported health centre [28, 63–65]. Between mid-December 2020 and early March 2021, 111 war-wounded patients were treated by MSF in Bambari [63]. Decreased patient consultations at MSF supported healthcare facilities have been noted, and access to certain areas of the prefecture by MSF teams has been blocked (personal communication MSF project Ouaka). Mortality might be even higher now than it was at the time of this survey.

Even prior to the current upsurge in conflict in late 2020, it was predicted that, 2.8 million people across CAR, more than half of the population, would require humanitarian assistance in 2021 [28]. Meeting the needs arising from CAR’s protracted crisis requires considerable, sustained, and effective humanitarian and development support across all domains. However, since 2011, only between 38 and 70% or UN funding targets were met by donors [12].

Ouaka is not unique in the challenges it faces [61], making it a reasonable assumption that the results of this survey might be generalisable to other regions of CAR. We have shared the results with governmental and non-governmental agencies working in Ouaka and across CAR for programming and advocacy. We will use these results of this study to advocate for the people of Ouaka and CAR, both within CAR and globally.

### Limitations

We cannot rule out underestimation of the true mortality as we were unable to include 4 communes due to insecurity in the area. It is possible that we have missed areas with worse living conditions and increased deaths due to violence and limited access to healthcare. The study is subject to limitations commonly associated with this study type including recall bias, reporting bias, and survival bias [34]. We relied on verbal reporting by the household head and causes of death were not verified through other sources. Non-specific symptoms such as fever and diarrhoea can have a wide differential diagnosis, particular in young children. Therefore, the reported causes of death may not reflect the exact contributors to mortality. The verbal reporting also resulted in a high proportion of unknown causes of death, many of which could be due to common morbidities as identified in the global burden of disease study [66] such as tuberculosis (TB) and HIV/AIDS with can have insidious clinical presentations. The global burden of disease data estimates TB, diarrheal diseases, lower respiratory tract infections, HIV/AIDS, neonatal disorders and malaria to be the most prominent causes of death [66].

Of note, the qualitative data arises from a single introductory question which asked what difficulties the household faces. While it has provided valuable data, a more comprehensive qualitative approach is required for a deeper understanding of challenges faced by the community. In addition, the responses to the open question were not recorded verbatim or audio recorded, which may have introduced a bias based on the surveyor’s interpretation of the household’s response or inter-surveyor variability in how responses were recorded. Translation from Sango to French by the surveyors may have introduced inaccuracies.

Lastly, we excluded buildings in settlements with less than 10 buildings from the sampling frame for feasibility reasons. Such settlements may be in rural remote areas, with less access to healthcare and therefore have a worse health status. By excluding them we may have underestimated the CMR.

### Need for further research

To get a better understanding of the national health situation, the planned country-wide survey will be undertaken as soon as feasible. Given the lack of other reliable data, security permitting, we will aim to conduct mortality surveys every 5–10 years thereafter for as long as our program run. In addition, complementary studies should be carried out to get more precise estimates of maternal mortality and its causes. Further research is needed to understand barriers to available care, health seeking behaviour and health literacy.

## Conclusion

While the region was not considered an acute humanitarian emergency at the time of the survey, we found high mortality estimates in excess of previous estimates, including a CMR which exceeds the emergency threshold [18–20, 34, 43, 44]. The reasons of death speak to poor living conditions, persistent violence and lack of access to healthcare and preventive measures.

The U5MR is also higher than previously estimated and nearing the emergency threshold but might still underestimate the true extent of deaths in children < 5 years. Violence continues to be a threat to life and to the physical, mental and social wellbeing of the civilian population despite the presence of MINUSCA forces and a reduction in fighting in the years prior to the study. The preventable and/or easily treatable illnesses of fever/malaria, diarrhoea, respiratory infections and measles are also leading causes of mortality, particularly amongst children < 5 years. Furthermore, the study showed that there are barriers to accessing healthcare, in particular distance to care and financial barriers. The fact that the majority of those that died reportedly sought care, might represent a troubling indicator for the quality of care available. The findings reinforce the need to increase access to free, proximate and good quality primary and secondary healthcare. The high MMR and neonatal mortality rate, the contribution of complications of induced abortions to maternal mortality, the high birth rate and the large proportion of pregnant women among women of reproductive age call for accessible reproductive healthcare including safe deliveries, contraception and safe abortion care.

The answer to our opening question provided a terrifying glimpse on challenges Central Africans face as part of their daily lives - even before the conflict intensified again in December 2020 - and are a reminder that improving health requires actions far wider than the provision of healthcare.

It appears that the challenges have changed little since 2018 when the mother of 11 from Ouaka foreboded that 1 day the “…*problems will have killed us all*”. While this study is only of one prefecture, many areas of CAR face similar challenges to Ouaka. If the results found in this study were generalisable across CAR, it would suffer one of the highest mortality rates in the world. An upsurge in violence across CAR, including in Ouaka, since December 2020, has likely further worsened the humanitarian situation. Each year, CAR is far from meeting its UN appeal for funding targets, allowing a silent crisis to continue. To reduce mortality in CAR, a comprehensive approach aiming to improve in basic living conditions and assure access to quality healthcare and preventive measures, underpinned by the achievement of peace, is urgently needed.

## Supplementary Information

**Additional file 1** Survey questionnaire in XML format, Retrospective mortality survey, Ouaka, CAR, 2020.

**Additional file 2 **Symptoms reported for deaths where the cause was not known (*N* = 48), Retrospective mortality survey, Ouaka, CAR, 2020.

## Data Availability

MSF has a managed access system for data sharing that respects MSF’s legal and ethical obligations to its patients to collect, manage and protect their data responsibility. Ethical risks include, but are not limited to, the nature of MSF operations and target populations being such that data collected are often highly sensitive. Data are available on request in accordance with MSF's data sharing policy (available at: http://fieldresearch.msf.org/msf/handle/10144/306501). Requests for access to data should be made to data.sharing@msf.org.

## Abbreviations

CAR: Central African Republic
CMR: Crude mortality rate
95% CI: 95% confidence interval
DEFF: Design effect
HIV: Human immunodeficiency virus
ICASEES: Institut Centrafricaine des Statistique et des Etudes Economique et Sociales *(Central African Institute for Statistics and Economic and Social Studies)*
IDP: Internally displaced person
IQR: Interquartile range
MINUSCA: Mission multidimensionnelle intégrée des Nations Unies pour la stabilisation en République centrafricaine *(United Nations Multidimensional Integrated Stabilization Mission in the Central African Republic)*
MMR: Maternal mortality ratio
MSF: Médecins sans Frontières
TB: Tuberculosis
U5MR: Under-five mortality rate (mortality rate in children under 5 years of age)
UN: United Nations

## References

[1] Médecins sans Frontières. Suffering mounts as armed groups return to Bambari. Available from: https://www.msf.org/suffering-mounts-armed-groups-return-bambari. Accessed 30 Oct 2020.

[2] United Nations Human Rights Office of the High Commissioner (OHCRH). Rights Committee: entrenched impunity and “infernal cycle of violence” at the heart of the dialogue with the Central African Republic. Available from: https://www.ohchr.org/EN/NewsEvents/Pages/DisplayNews.aspx?NewsID=25665&LangID=E. Accessed 30 Oct 2020.

[3] Lombard L (2016). State of rebellion: violence and intervention in the Central African Republic.

[4] Welz M (2014). Briefing: crisis in the Central African Republic and the international response. Afr Aff.

[5] Human Rights Watch. Central African Republic - Events of 2019. Available from: https://www.hrw.org/world-report/2020/country-chapters/central-african-republic. Accessed 3 Sept 2020.

[6] International Crisis Group. Report 277/Africa; Making the Central African Republic’s Latest Peace Agreement Stick. Available from: https://www.crisisgroup.org/africa/central-africa/central-african-republic/277-making-central-african-republics-latest-peace-agreement-stick. Accessed 30 Oct 2020.

[7] United Nations Security Council. Letter dated 14 February 2019 from the secretary-general addressed to the president of the security council; political agreement for peace and reconciliation in the Central African Republic; S/2019/145. United Nations security council; 2019. ssss.

[8] United Nations Development Programme (UNDP). Human Development Data (1990–2018). Available from: http://hdr.undp.org/en/data. Accessed 30 Oct 2020.

[9] Fund for peace. Fragile states index. Available from: https://fragilestatesindex.org/data/. Accessed 30 Oct 2020.

[10] Central African Republic - A state of silent crisis. Médecins Sans Frontières; 2011. Available from : https://www.msf.org/sites/msf.org/files/2018-06/A_State_of_Silent_Crisis_EN.pdf

[11] World Health Organization. Humanitarian Health Action; A silent crisis. Available from: https://www.who.int/hac/crises/caf/a_silent_crisis_moh_meeting/en/. Accesssed 11 Nov 2020.

[12] Financial tracking service. Central African Republic 2019 (Humanitarian response plan): OCHA. Available from: https://fts.unocha.org/appeals/674/summary. Accessed 2 Aug 2020.

[13] United Nations Development Programme (UNDP). Life expectancy at birth (years). Available from: http://hdr.undp.org/en/indicators/69206. Accessed 30 Oct 2020.

[14] UNDP. Human Development Reports; Median age (years). Available from: http://hdr.undp.org/en/indicators/47906. Accessed 21 Oct 2020.

[15] UNFPA, World Health Organization, UNICEF, World Bank Group, the United Nations population division (2019). Trends in maternal mortality 2000 to 2017: estimates by WHO, UNICEF, UNFPA, World Bank Group and the United Nations population division.

[16] The World Bank. Birth rate, crude (per 1,000 people) - Central African. Available from: https://data.worldbank.org/indicator/SP.DYN.CBRT.IN?locations=CF. Accessed 30 Oct 2020.

[17] UN Inter-agency Group for Child Mortality Estimation (IGME) (2019). Levels & Trends in Child Mortality: Report 2019, Estimates developed by the United Nations inter-agency Group for Child Mortality Estimation.

[18] The World Bank. Death rate, crude (per 1,000 people) - Central African Republic. Available from: https://data.worldbank.org/indicator/SP.DYN.CDRT.IN?locations=CF. Accessed 10 Nov 2020.

[19] Ministère de la Santé et de la Population, Institut Centrafricain de Statistique et Etudes Economiques et Sociales. Résultats de l'enquête nutritionnelle nationale (SMART) RCA [Results of the national nutritional survey (SMART) CAR]. 2018.

[20] UNICEF. Résultats préliminaires de l’enquête nutritionnelle SMART, 2019 [Preliminary results of the SMART nutritional survey, 2019]. 2020.

[21] Médecins Sans Frontières. Rapport évaluation rapide nutrition et mortalité rétrospective - Préfecture de la Basse-Kotto, District Sanitaire d’Alindao-Mingala, Juin-Juillet 2019 [Report of the rapid asssesment of nutrition and retrospective mortality - Prefecture of Basse-Kotto, Health district of Alindao-Mingala, June-July 2019](Internal Report). Bangui: MSF; 2019.

[22] Agence Humanitaire Africaine. SMART rapide dans la commune de Mobaye, Sous-préfecture de Mobaye, Préfecture de la Basse Kotto, République Centrafricaine du 15 au 18 Mars 2018 [Rapid SMART in the commune of Mobaye, sub-prefecture of Mobaye, Prefecture of Basse Kotto, Central African Republic, from 15 to 18 March, 2018]. Bangui: AHA; 2018.

[23] Agence Humanitaire Africaine. SMART rapide dans la localité de Langandi, commune de Mbelima, Sous-préfecture de Mobaye, Préfecture de la Basse Kotto, République Centrafricaine du 15 au 18 Mars 2018 [Rapid SMART in the locality of Langandi, commune of Mbelima, sub-prefecture of Mobaye, Prefecture of Basse Kotto, Central African Republic, from 15 to 18 March, 2018]. Bangui: AHA; 2018.

[24] Agence Humanitaire Africaine. SMART Rapide dans la commune de Ouambé, Sous-Préfecture de Zangba, Préfecture de la Basse Kotto, du 15 au 24 février 2018 [Rapid SMART in the commune of Ouambé, sub-prefecture of Zangba, Prefecture of Basse Kotto, from 15 to 24 February, 2018]. Bangui: AHA; 2018.

[25] Institut Centrafricaine des Statistique et des Etudes Economique et Sociales. Population RCA par commune 2003–2019 [CAR population by commune 2003-2019].

[26] Organisation Internationale pour les Migrations (OIM). République Centrafricaine (RCA) Matrice de Suivi des Déplacements (DTM) Rapport 9; Avril 2020 [Central African Republic (CAR) Displacement Tracking Matrix (DTM) Report 9; April 2020]. Bangui: OIM; 2020. Available from: https://reliefweb.int/report/central-african-republic/rca-matrice-de-suivi-des-d-placements-dtm-rapport-9-avril-2020.

[27] Organisation Internationale pour les Migrations (OIM). République Centrafricaine (RCA) Matrice de Suivi des Déplacements (DTM) Rapport 7; Juillet 2019 [Central African Republic (CAR) Displacement Tracking Matrix (DTM) Report 9; July 2019]. Bangui: OIM; 2019. Available from : https://reliefweb.int/report/central-african-republic/rca-matrice-de-suivi-des-d-placements-dtm-rapport-7-juin-2019.

[28] OCHA. Central African Republic Situation Report 25 Feb 2021. Available from: https://reliefweb.int/updates?search=primary_country.iso3:caf%20AND%20ocha_product:(%22Humanitarian%20Bulletin%22%20OR%20%22Situation%20Report%22%20OR%20%22Flash%20Update%22)%20AND%20source:OCHA#content. Accessed 26 Feb 2021.

[29] UNICEF (2019). Central African Republic humanitarian situation report January 2019.

[30] Human Rights Watch. Central African Republic: Don’t Reward Warlords 2019. Available from: https://www.hrw.org/news/2019/04/24/central-african-republic-dont-reward-warlords. Accessed 11 Nov 2020.

[31] Médecins Sans Frontières. Project Situation Reports; Bambari; January–April 2019 (Internal Reports). Bambari: MSF; 2019.

[32] United Nations Office for the Coordination of Humanitarian Affairs (OCHA). République Centrafricaine; aperçu de l’état des formations sanitaires (FOSA) [Central African Republic; overview of the state of health facilities]. Available from: https://app.powerbi.com/view?r=eyJrIjoiYmY4ZGEzZjctNTEwNS00N2NlLWJmYjEtYTdhZjZiM2RkMDY3IiwidCI6IjBmOWUzNWRiLTU0NGYtNGY2MC1iZGNjLTVlYTQxNmU2ZGM3MCIsImMiOjh9. Accessed 23 Oct 2020.

[33] ENA Software for SMART. Available from: https://smartmethodology.org/survey-planning-tools/smart-emergency-nutrition-assessment/. https://smartmethodology.org/survey-planning-tools/smart-emergency-nutrition-assessment/

[34] Checchi F, Roberts L (2005). Interpreting and using mortality data in humanitarian emergencies; a primer for non-epidemiologists.

[35] Carrion Martin AI, Bil K, Salumu P, Baabo D, Singh J, Kik C (2014). Mortality rates above emergency threshold in population affected by conflict in north Kivu, Democratic Republic of Congo, July 2012-April 2013. PLoS Negl Trop Dis.

[36] Robinson E, Crispino V, Ouabo A, Soung Iballa FB, Kremer R, Serbassi ME, van Lenthe M, Carrion Martin AI (2019). Mortality and health survey, Walikale, Democratic Republic of the Congo, 2017: an example of the use of survey data for humanitarian program planning. Confl Heal.

[37] OCHA Centre for Humanitarian Data. Central African Republic - Subnational Administrative Boundaries Available from: https://data.humdata.org/dataset/central-african-republic-administrative-boundaries. Accessed 30 Novr 2019.

[38] Digital Globe, Ecopia (2017). Technical specification Ecopia building footprints powered by DigitalGlobe, September 2017. Digital Globe.

[39] Harvard Humanitarian Initiative. About Kobo Toolbox. Available from: https://www.kobotoolbox.org/. Accessed 29 Oct 2020.

[40] StataCorp (2017). Stata Statistical Software: Release 15.

[41] Hsieh HF, Shannon SE (2005). Three approaches to qualitative content analysis. Qual Health Res.

[42] Morgan DL (1993). Qualitative content analysis: a guide to paths not taken. Qual Health Res.

[43] UNHCR. Mortality surveillance threshold. Available from: https://emergency.unhcr.org/entry/85919/mortality-surveillance-threshold. Accessed 10 Nov 2020.

[44] Epicentre (2006). Rapid Health Assessment of Refugee or Displaced Populations.

[45] Taylor WR, Chahnazarian A, Weinman J, Wernette M, Roy J, Pebley AR, Bele O, Ma-Disu M (1993). Mortality and use of health services surveys in rural Zaire. Int J Epidemiol.

[46] Becker SR, Diop F, Thornton JN (1993). Infant and child mortality in two counties of Liberia: results of a survey in 1988 and trends since 1984. Int J Epidemiol.

[47] MINUSCA (2015). Press release: Herve Ladsous announces establishment of a weapon-free zone in Bambari: MINUSCA.

[48] Nareeba T, Dzabeng F, Alam N, Biks GA, Thysen SM, Akuze J (2021). Neonatal and child mortality data in retrospective population-based surveys compared with prospective demographic surveillance: EN-INDEPTH study. Popul Health Metrics.

[49] Bearak J, Popinchalk A, Ganatra B, Moller AB, Tunçalp Ö, Beavin C, Kwok L, Alkema L (2020). Unintended pregnancy and abortion by income, region, and the legal status of abortion: estimates from a comprehensive model for 1990-2019. Lancet Glob Health.

[50] UN Inter-agency Group for Child Mortality Estimation. Methods; child mortality. Available from: https://childmortality.org/methods. Accessed 8 Mar 2021. 10.1371/journal.pone.0101112 PMC4094389 25013954

[51] UN Inter-agency Group for Child Mortality Estimation. Central African Republic; under-five mortality rate; source data. Available from: https://childmortality.org/data. Accessed 8 Mar 2021.

[52] United Nations Department of Economic and Social Affairs, Population Dynamics. World Population Prospects; data sources; Central African Republic. Available from: https://population.un.org/wpp/DataSources/140. Accessed 8 Mar 2021.

[53] ICASEES. MICS6-RCA; Enquête par grappes à indicateurs multiples 2018-2019, Rapport final des resultats de l'enquete [MICS6-CAR; Multiple Indicator Cluster Survey 2018-2019, final report of survey results]. Bangui: ICASEES; 2021.

[54] Wol P, Kay C, Roberts L (2021). Interviews about attended births appear to be deceptive in CAR: Are the population saying what they think NGO’s want to hear?. Confl Heal.

[55] Bradley S. When quality matters: linking the reliability of demographic and health survey data to biases in international mortality, fertility, and family planning estimates: UC Berkeley; 2016.

[56] Checchi F, Warsame A, Treacy-Wong V, Polonsky J, van Ommeren M, Prudhon C (2017). Public health information in crisis-affected populations: a review of methods and their use for advocacy and action. Lancet..

[57] International Red Cross, Columbia University (2018). Baseline Assessment Report; Birth and death rate information, Ouham-Pende areas served by IRC (Internal Report). IRC.

[58] Caleo GM, Sy AP, Balandine S, Polonsky J, Palma PP, Grais RF (2012). Sentinel site community surveillance of mortality and nutritional status in southwestern Central African Republic, 2010. Popul Health Metrics.

[59] Perales NA, Wei D, Khadka A, Leslie HH, Hamadou S, Yama GC, Robyn PJ, Shapira G, Kruk ME, Fink G (2020). Quality of clinical assessment and child mortality: a three-country cross-sectional study. Health Policy Plan.

[60] Garry S, Checchi F (2020). Armed conflict and public health: into the 21st century. J Public Health (Oxf).

[61] OCHA. Apercu des besoin humanitaires, République centrafricaine [Overview of humanitarian needs, Central African Republic]. Bangui: OCHA; 2020.

[62] OCHA. République centrafricaine Plan de Réponse Humanitaire 2021 [Central African Republic Humanitarian Response Plan 2021]. Bangui: OCHA; 2020.

[63] Médecins Sans Frontières. People, medical facilities hit during violence in southern CAR. Available from: https://www.msf.org/people-medical-facilities-hit-during-violence-southern-car. Accessed 1 Mar 2021.

[64] Médecins Sans Frontières. Shooting shows civilians continue to pay high price for the perpetual cycle of violence in CAR. Available from: https://www.msf.org/shooting-incident-near-bambari-car-kills-people-including-msf-staff. Accessed 1 Mar 2021.

[65] OHCRH. CAR: Violations of human rights and international humanitarian law must be punished to prevent ongoing violence and conflict. Available from: https://www.ohchr.org/EN/NewsEvents/Pages/DisplayNews.aspx?NewsID=26664&LangID=E. Accessed 26 Feb 2021.

[66] Global burden of 369 diseases and injuries in 204 countries and territories, 1990–2019: a systematic analysis for the Global Burden of Disease Study 2019 [supplementary appendix 2]. Lancet. 2020;396(10258):1204–22. 10.1016/S0140-6736(20)30925-9 PMC7567026 33069326

