# Supplementary material for: Mortality beyond emergency threshold in a silent crisis– results from a population-based mortality survey in Ouaka prefecture, Central African Republic, 2020
Source: Confl Health. 2021 Jun 30;15:50. doi: 10.1186/s13031-021-00385-2 (PMC8243074; doi:10.1186/s13031-021-00385-2)
Supplement: Supplementary file 2 — Additional file 2 Symptoms reported for deaths where the cause was not known (N = 48), Retrospective mortality survey, Ouaka, CAR, 2020. [file 13031_2021_385_MOESM2_ESM.docx]

**Additional file to: Eve Robinson et al.: Mortality beyond emergency threshold in a silent crisis– results from a population-based mortality survey in Ouaka prefecture, Central African Republic, 2020**

Additional file 1: Symptoms reported for deaths where the cause was not known (N=48), Retrospective mortality survey, Ouaka, CAR, 2020.

| **Symptoms or signs, or other reported cause of death** | **Number of cases** | **Age range (years unless specified)** |
| --- | --- | --- |
| Ascites | 1 | 20 |
| Sudden death/ death in sleep | 3 | 25—43 |
| Swelling of stomach and legs | 1 | 25 |
| Swelling of legs, diarrhoea, weight-loss for several months | 2 | 10—32 |
| Swelling, vomiting, jaundice | 1 | 6 |
| Stomach swelling and fever | 1 | 14 |
| Stomach and body swelling for several months | 1 | 47 |
| Oedema and jaundice | 3 | 3—12 |
| Oedema and headache | 2 | 27—60 |
| Generalised oedema for one month | 1 | 20 |
| Swelling of stomach and legs, diarrhoea, vomiting blood for several months | 1 | 30 |
| History of chest pain and a heart problem. vomiting and weight loss prior to death | 1 | 15 |
| Vomiting blood | 2 | 19—35 |
| Vomiting blood with cough | 1 | 30 |
| Vomiting blood, nose bleeds, swelling of body | 1 | 2 |
| Abdominal pain and headache | 1 | 28 |
| Constant cough with growth on chest | 1 | 78 |
| Paralysis of lower limbs for several months followed by vomiting | 1 | 33 |
| Paralysis | 1 | 30 |
| Depression | 1 | 21 |
| Mental health | 1 | 40 |
| Stopped speaking and eating | 1 | 20 |
| Sorcery | 3 | 28—40 |
| Headache and dizziness | 1 | 40 |
| 5 year history of headaches and dizziness | 1 | 70 |
| Refusal to eat for week before death. 5 year history of worsening back pain | 1 | 77 |
| Did not fully recover from an assault one year before | 1 | 65 |
| Previous fracture of knee, did not fully recover | 1 | 70 |
| Sudden death in sleep preceded by swelling of legs and parasitic infection | 1 | 51 |
| Somnolence and fevers for 2 weeks. Mental health alteration | 1 | 65 |
| Stomach of swelling, did not pass stools, fever for 5 days | 1 | 2 |
| Swollen stomach for 2 weeks. Parasitic infection | 1 | 47 |
| Haematuria, vomiting and fever for 1 day | 1 | 7 months |
| 10 year history of paralysis but no specific symptoms before death | 1 | 62 |
| Uvula removal | 1 | 60 |
| Gynaecological problem | 1 | 57 |
| Weight loss, and lots of pain preventing him from working | 1 | 35 |
| Unknown | 2 | 1—66 |
